# Phytosterols in rice bran and their health benefits

**DOI:** 10.3389/fnut.2023.1287405

**Published:** 2023-10-12

**Authors:** Zhaoguo Liu, Xiaoxiao Liu, Zheng Ma, Tianzhu Guan

**Affiliations:** ^1^Changchun Institute of Technology, Changchun, China; ^2^School of Food Science and Engineering, Yangzhou University, Yangzhou, China; ^3^Department of Thoracic Surgery, Qilu Hospital of Shandong University, Jinan, China

**Keywords:** rice bran, plant terpenoids, phytosterols, health benefits, processing technology, stability evaluation

## Abstract

With the continuous technological innovation in the high-value utilization of rice bran byproducts, rice bran oil retains a higher concentration of beneficial components such as a well-balanced composition of fatty acids and abundant phytosterols. This makes it a highly nutritious and healthy vegetable oil. This review provides an overview of the advancements made in separating, purifying, and processing phytosterols in rice bran oil. The review also introduces techniques for assessing the stability of rice bran oil. Moreover, the review emphasizes the nutritional value of phytosterols found in rice bran oil, highlighting their various health benefits, including their anticancer, anti-inflammatory, anti-allergic, antibacterial, cholesterol-lowering, skin-protective, anti-obesity, anti-diabetic, neuroprotective, gastroprotective, and immune-enhancing effects. Attaining a comprehensive understanding of the research progress made in phytosterols derived from rice bran oil can offer valuable guidance for the efficient utilization of rice bran.

## 1. Introduction

Rice bran is the part of rice removed during the process of dehusking and milling, which transforms rough rice into polished rice (1, 2). It refers to the brownish outer layer of rice grains, including the outer husk, bran layer, aleurone layer, seed coat, and endosperm (3). It is also known as “rice husk” or “rice bran,” and typically constitutes about 8% of the total weight of the rice grain (4). As a byproduct of the rice milling process, rice bran often contains rice germ and broken rice. Furthermore, the composition of rice bran can be significantly influenced by different rice varieties and the precision of polished rice (5). Generally, regular rice bran contains around 34 to 62% carbohydrates, 11 to 15% protein, 15 to 20% fat, 7 to 11% dietary fiber, and 8 to 12% ash (6). Additionally, several bioactive compounds are found, such as tocopherols, tocotrienols, phenolic acids, flavonoids, oryzanol, octacosanol, α-lipoic acid, squalene, and ceramides, among others (7).

During the processing of brown rice, the mechanical actions disrupt the cellular structure of the rice bran layer, causing lipases from the endosperm to disperse into the rice bran (8). When exposed to the oil in the bran, hydrolysis reactions take place, leading to the rapid hydrolytic rancidity of rice bran, accompanied by producing a pungent odor that affects its development and utilization (9). Research has shown that rice bran requires stabilization techniques within 6 h to deactivate the lipases, in order to achieve its effective utilization and storage. Currently, stabilization techniques for rice bran are adopted widely, including dry heat treatment, low-temperature storage, microwave heating inactivation, chemical stabilization, ohmic dielectric heating, radiation treatment, extrusion puffing, enzymatic treatment, among others (10). Among these methods, dry heat treatment is the most commonly used for enzyme deactivation, although it may also affect activities of rice bran proteins (11). Nutrient loss can be easily caused by the extrusion puffing method, while chemical stabilization may result in the retention of chemical residues, so this method is less commonly employed (12).

Rice bran has an oil content ranging from 12 to 24%, and based on its lipid content, it can be categorized into three types: full-fat rice bran, low-fat rice bran, and defatted rice bran (13). Full-fat rice bran is commonly referred to as rice bran, which has not undergone any oil extraction process. It retains its natural oil content. Low-fat rice bran is the type of rice bran obtained after extracting rice bran oil, and it typically contains around 7% of oil. Defatted rice bran, also known as rice bran meal, is the residue left after extracting rice bran oil using organic solvents (14). It contains less than 1% of oil. Moreover, in actual production, rice bran can be further classified into “rough rice bran” and “polished rice bran” based on the number of times of rice milling processes and different techniques employed (15). Rough rice bran, with fewer milling processes, has a higher lipid content, making it suitable for oil extraction purposes. On the other hand, polished rice bran has lower lipid content but is rich in protein, making it suitable for the development of nutritious food products (16).

In order to preserve and retain the various bioactive compounds abundant in rice bran and avoid their destruction, the processing techniques of rice bran oil have been widely researched (17). These techniques include mechanical pressing, solvent extraction, supercritical CO_2_ extraction, membrane separation technology, and more. Rice bran oil has a balanced fatty acid composition, containing approximately 22% saturated fatty acids, 41% monounsaturated fatty acids, and 37% polyunsaturated fatty acids (18). The main components of these fatty acids are palmitic acid, oleic acid, and linoleic acid, respectively, with their proportions closely matching the dietary fatty acid recommendations of the World Health Organization (WHO) at 1:1.5:1 (19). Additionally, rice bran oil contains high levels of phytosterols, tocopherols, and tocotrienols, with concentrations reaching up to 10,500 mg/kg, 15,300 mg/kg, and 205.7 mg/kg, respectively, far surpassing those found in other plant oils. As a result, rice bran oil has been utilized in the development of functional blended oils, frying oils, and other products (20).

In addition to being used for oil extraction, rice bran has further expanded its applications in the food, health supplement, and pharmaceutical industries by extracting high-nutrition and high-economic-value products from the defatted byproduct, rice bran meal (21). These extracted products include polysaccharides, dietary fiber, proteins, phytic acid, inositol, and other valuable components. Numerous studies have demonstrated that rice bran extracts possess various nutritional and health benefits (22). For instance, rice bran polysaccharides exhibit anti-tumor, immune-enhancing, and blood glucose-lowering effects. Rice bran dietary fiber, being the highest polysaccharide content, can be added to various food products such as pasta and baked goods to improve their texture and nutritional value. Rice bran protein contains all essential amino acids and is easily digestible and absorbed by the human body. Further hydrolysis of rice bran protein yields biologically active peptides with rich nutritional properties (23). Research has shown that these peptides have effects such as lowering blood pressure, reducing cholesterol levels, anti-cancer properties, antimicrobial activities, and alleviating diabetes symptoms (24).

Overall, the existing research has provided a comprehensive understanding of rice bran dietary fiber, protein, and oil. There have also been comprehensive summarizations of rice bran oil-derived compounds such as oryzanol, tocopherols, and tocotrienols, which are present in significant quantities. However, compared to other plant oils, the remarkable benefits of plant sterols found in rice bran have received less attention in the literature. This review focuses on rice bran, a byproduct of rice processing, and review the current research and application status of the retained nutrients and accompanying compounds, particularly plant sterols, in the deep processing of rice bran oil.

## 2. Chemical structures of phytosterols

**Figure 1:**
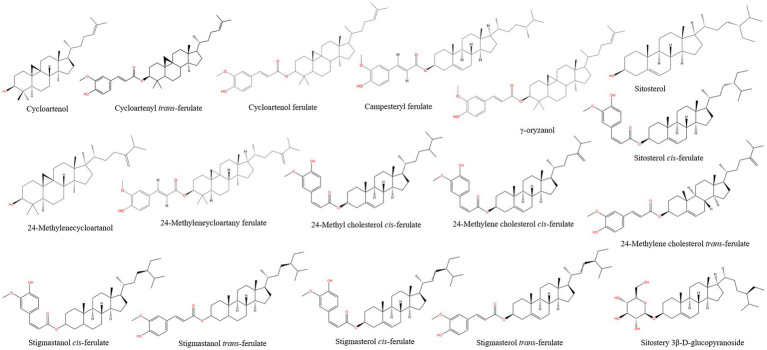
Chemical structures of phytosterols from rice bran.

Phytosterols, generally known as plant steroids, have similar structural and biological functions to cholesterol (25). With regard to health-promoting effects on human beings, it has been claimed from several scientific literatures that phytosterol presented remarkable anti-oxidant, anti-polymerization, anti-inflammatory, anti-tumor and other biological activities. At present, it is reported that more than 200 different types of phytosterols have been identified in a wild range of plant species with trace levels (26, 27). The chemical structures of identified phytosterols compounds from rice bran are displayed in Figure 1. Naturally, the basic skeleton of phytosterols is cyclopentane polyhydrophenanthrene with a hydroxyl group at C-3 position and an 8 to 10 carbon atoms side chain at C-17 position, which could significantly influence the physiochemical properties and biological properties of phytosterols. With regard to the number of methyl groups at the C-4 position, phytosterols can be divided into three categories: 4-demethyl sterols, 4a-monomethyl sterols, and lophenol; 4,4-dimethyl sterols. Given their inherent molecular structure, the main differences are that phytosterols usually have a methyl or ethyl group at the C-24 position, and some common phytosterols have an unsaturated double bond at the C-22 position. Moreover, the poor oil-solubility and water-solubility are contributed to the hydroxyl and hydrocarbon side chains at C-3 and C-17 (28). However, humans cannot synthesis phytosterols, all phytosterols in human blood and tissues are derived from diet (29). Due to their diverse physiological effects, phytosterols have great potential to use in functional foods, supplements, and pharmaceutical products designed to enhance human health. Therefore, the development and utilization of the phytosterols from rice bran is necessary.

## 3. Technologies for phytosterols extraction and purification

The main traditional methods for phytosterols extraction from rice bran are cold-pressure extraction (CPE) (30) and solvent extraction (SE) (31). Nevertheless, traditional methods exhibit shortcomings in terms of time consumption, energy inefficiency, limited economic viability, and environmental contamination (32). Thus, researchers have reported novel techniques for extracting phytosterols, such as ultrasonication assisted extraction (33), supercritical fluid extraction (SFE) (34), subcritical extraction (SUBE) (35), and ohmic heating-aided mechanical extraction (36). The yields of γ-glutamin from rice bran obtained by SE, SFE, and SUBE and the concentrations in the extracts are presented in Table 1.

**Table 1:** The extraction yields of phytosterols in rice bran obtained by SE, SFE, and SUBE.

Extraction method	Solvent	Parameters/Condition	Yield	References
SFE	CO_2_	65°C; 48 Mpa; 4 h; 0.45 mL CO_2_/min	11371.79 mg/kg of dry rice bran	(37)
	CO_2_	48.9°C; 23.9 Mpa; 29.8 g CO_2_/min	36,000 mg/kg of rice bran	(38)
	CO_2_	43°C; 5,420 psi; 60 min	2,730 μg/g of rice bran	(34)
	CO_2_	350 bar; 313 K; 4 h	84.9%	(39)
	CO_2_	41.4 MPa; 60°C; 2.5 L CO_2_/min	3104.8 mg/100 g of dry rice bran	(40)
	CO_2_	30 MPa; 303 K; 270 min; 10 g CO_2_/min	39% (w/w) of rice bran oil	(41)
SUBE	Dimethyl ether	0.8 MPa; 60°C; 30 min	8128.51 mg/100 g of rice bran oil	(42)
	Dimethyl ether	1.8 MPa; 70°C; 60 min	1213.64 mg/100 g of rice bran oil	(35)
	Dimethyl ether	<1 MPa; 30 min	924.51 mg/100 g of defatted rice bran	(43)
SE	Ethanol	From 32°C to 55–60°C; 24 h; 100:1 (w/w); 200 rpm	9414.02 mg/kg of dry rice bran	(37)

Supercritical fluid is any substance at a temperature and pressure above its critical point, where distinct liquid and gas phases do not exist (44). SFE often uses CO_2_ and water as solvents. In the study of Imsanguan et al., three methods (SC-CO_2_, SE, and Soxhlet extraction) were compared for the γ-oryzanol extraction and found that the maximum extraction rate of 11371.79 mg/kg of dry rice bran was achieved by SC-CO_2_ (37). Understanding the solubility of γ-oryzanol in SC-CO_2_ is the basis for optimization of the extraction conditions for SC-CO_2_. Bitencourt et al. found that the process parameters, such as CO_2_ flow rate, pressure and temperature, plays an important role in SC-CO_2_ extraction. Under the optimal conditions, the solubility of γ-oryzanol at 60°C/400 bar was close to 1 g/kg in CO_2_ (45). For instance, γ-oryzanol was reported to have a high recovery (39% (w/w)) using SC-CO_2_ at 30 MPa, 303 K, 270 min and 10 g CO_2_/min (41). Considering the techno-economy of SC-CO_2_, Kayathi et al. realized SC-CO_2_ extraction of γ-oryzanol from rice bran with low pressure operation (23.9 Mpa), which yielded up to 36,000 mg/kg of dry rice bran (38). In another study conducted by Chen et al., using SC-CO_2_ to extract rice bran oil from rice bran flour, followed by the concentration and isolation of γ-oryzanol by column partition purification (N-hexane with ethyl acetate in volume ratios of 90:10) can get high purity γ-oryzanol (39).

Subcritical liquified dimethyl ether (SUBLDME) extraction will consume less energy than SC-CO_2_, while also reducing greenhouse gas emissions. Compared to SC-CO_2_, SUBLDME yielded more γ-oryzanol (4865.25 mg/100 g of rice bran) (42). SUBLDME often uses propane, butane, dimethyl ether and water as solvents. Among them, dimethyl ether has a low boiling point (−24.8°C) and is not easy to remain in the extract. Dimethyl ether has been authorized by the European Food Safety Authority (EFSA) as a safe extraction solvent for the production of foodstuffs and food ingredients (43). Moreover, there is considerable experimental data suggesting that subcritical butane- and propane-extracted rice bran oil had high oxidative stability (46). Therefore, the use of subcritical solvents to create added value to agricultural wastes through the extraction of high-value food products is one of the strategies to reduce CO_2_ greenhouse gas emissions.

**Figure 2:**
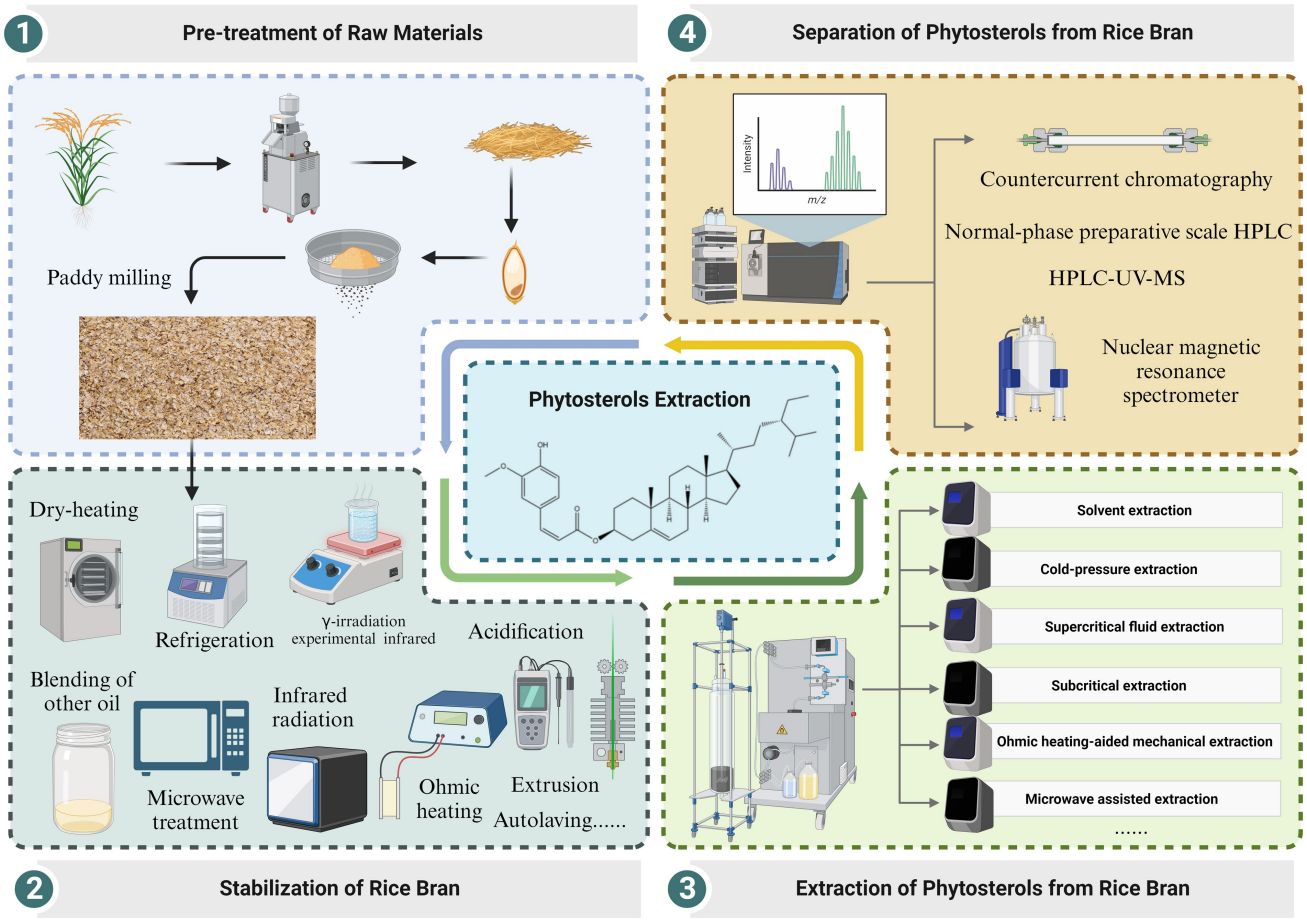
Extraction process of phytosterols from rice bran.

It is noteworthy that trace components may remain in the γ-oryzanol extracted from rice bran, such as cycloartenyl ferulate (CAFA) and campesteryl ferulate. Therefore, further isolation and purification of the extracted γ-oryzanol is also required. Currently, the commonly used separation and purification methods include countercurrent chromatography (CCC) (47) and normal-phase preparative scale high performance liquid chromatography (HPLC) (48). Recent evidence suggests that γ-oryzanol is separated by the preparative recycle HPLC system using a combination of octadecylsilane (ODS) silica and the purity of γ-oryzanol exceeds 99% (49). In addition, Sawada et al. used high performance liquid chromatography-ultraviolet-mass spectrometry (HPLC-UV-MS) and nuclear magnetic resonance (NMR) for the separation and purification of the isomerized substance of γ-oryzanol (50). The extraction process of phytosterols from rice bran are summarized in Figure 2.

## 4. Stability of phytosterols from rice bran

Generally, the processing of phytosterols extraction starts from stabilization of rice bran. And then the stabilized rice bran was extracted by a number of extraction and separation methods. Rice bran is composed of numerous bioactive compounds, such as tocopherols, γ-oryzanol, anthocyanins, and flavonoids, which mainly contributed to the stability of rice bran. Among them, tocopherols could protect cell membranes, increase stability, and inhibit lipid peroxidation by acting as a trap for lipid peroxyl radicals. But tocopherols are highly susceptible to degradation at high temperatures. A strong relationship between combined bioactive and antioxidant capacity has been reported in the literature (51). They identify that high activity against free radicals and strong antioxidant capacity of tocopherols at lower concentration (0.04%). γ-oryzanol is second only to tocopherols in antioxidant power. When the ratio of rice bran to corn oil was 1:5 and the heating temperature was 180°C, the 2,2-diphenyl-1-picrylhydrazyl (DPPH) loss of corn oil decreased slowly, the *p*-anisidine value and CDA of corn oil increased the slowest, and corn oil stability was significantly improved during storage after recovery. Furthermore, BoRa Yi et al. suggested that the probable cause was the transfer of some of the γ-oryzanol from rice bran to corn oil (52). It is worth noting that a large number of studies have focused on the concentration of γ-oryzanol and tocopherols. However, the synergistic effect of antioxidant capacity depends not only on the concentration but also on the ratio. It was shown that the formation of FFA and secondary oxidation products was significantly inhibited while the optimal ratio of γ-oryzanol and tocopherols was 8:1, with the oxidative stability index of rice bran oil reached 10.26 h (53). Similarly, γ-oryzanol has a certain inhibitory effect on the degradation of tocopherols (54). When the concentration of tocopherols in rice bran oil was 500 mg/kg, higher concentrations of γ-oryzanol did not inhibit the degradation of tocopherols. In contrast, the higher concentration of γ-oryzanol, the more pronounced, respectively, was the inhibition of tocopherols degradation.

As a result, various methods of stabilizing rice bran have emerged, such as heat treatment, extrusion, γ-irradiation, refrigeration and acidification. Among them, the commonly used heat treatment methods mainly include dry-heating (DH), microwave heating (MH), ethanol vapor (EV), ohmic heating, and so on. Kim et al. (11) compared the storage stability of rice bran after DH, FDDH, MH, AC, and EV treatments. It was found that the FFA content of the heat-treated rice bran did not change significantly compared to that of the untreated group (FFA content gradually increased from 2.14 to 19.81%) during the storage period (24 weeks), and all of them were effective in stabilizing the oxidative rancidity of the rice bran, with DH being the most effective in delaying the formation of FFA. In addition, Bruscatto et al. (55) placed rice bran oil at 100°C and 180°C to determine the changes in its α-, (γ + β)-, and δ-tocopherol content with time, and the results of the experiment confirmed that the degradation of tocopherols increased significantly with increasing heating temperature. The highest percentage of α-tocopherol in rice bran oil was lost by 26.08% after heating at 100°C for 432 h, while it was no longer detected at 180°C for 240 h. The results showed that α-tocopherol was not detected in rice bran oil. The contents of (γ + β)-tocopherol in rice bran oil was 79.9 mg/100 g, and δ-tocopherol was 6.4 mg/100 g, respectively, when heated at 100°C for 432 h. At 180°C for 432 h, the contents of (γ + β)-tocopherol were 1.4 mg/kg and δ-tocopherol were 0.3 mg/kg. Srisaipet and Nuddagul found that the stability of rice bran oil was related to the content of γ-oryzanol in addition to the temperature during heat treatment (56). γ-oryzanol can react with KOH to form carbonyl compounds due to the breaking of double bonds and oxidation, which are oxidized to low molecular weight fatty acids during heating and lost through volatilization. In addition to the commonly used dry heat treatment, an infrared (IR) stabilization method to prevent rancidity of rice bran was developed by Yılmaz et al. (57). The FFA content of rice bran stabilized at 600 W IR power for 5 min remained below 5% for 165 days. There was no significant change in γ-oryzanol content or fatty acid composition in stabilized rice bran compared to raw rice bran. Therefore, IR can be used as a medium to inactivate lipase and extend the shelf life of rice bran.

## 5. Challenges in the extraction of phytosterols from rice bran

Rice bran undergoes hydrolytic rancidity owing to the contact between lipids and active enzymes. Among them, lipase causes hydrolysis of rice bran oil to produce free fatty acids (FFA). This hydrolytic rancidity affects the flavor and odor of rice bran, thus limiting further development of rice bran. Conventional alkaline refining deacidification leads to loss of crude oil as well as other bioactive components (58, 59). There are a number of novel alternative methods (e.g., fractionation and enzymatic) that can effectively remove FFA while also stabilizing the antioxidants in rice bran. Dunford et al. focused on the potential of continuous countercurrent SC-CO_2_ fractionation for the deacidification of rice bran oil. The results showed that fractionation at a low pressure of 138 Bar and a high temperature of 80°C effectively removed FFA from rice bran oil without any oryzanol loss in the extract (60). According to the literature (61), when the CO_2_ filling pressure was 8.0 MPa, the FFA content of rice bran oil decreased to 1.1% and the phytosterol conversion reached 93.2%. Moreover, Xu et al. proposed and developed a tandem continuous flow reactor, which is used to deacidify rice bran oil and product functional oils. Compared to commercial Novozym 435, the tandem continuous-flow enzymatic reactor removed 91.4% of FFA and increased phytosterol esters and diacylglycerol in rice bran oil by 9- and 12-fold, respectively. Based on this, the research team innovated the method of removing FFA. They prepared a nanomagnetic Fe_3_O/SiOx-g-P lipase (magnetic enzyme) by immobilizing CALB on Fe_3_O4/SiOx-g-P polymer carrier. Using this as a catalyst, FFA from rice bran oil were used to esterify phytosterols. The FFA content in rice bran oil was reduced from 16.0 to 2.4%, and the conversion of phytosterols was 85.7% (62). In addition, the retention rate of γ-oryzanol is more than 40% higher than that of traditional alkaline refining method (63). In order to improve the reuse and value-added conversion of crude high acid rice bran oil, enzymes are often employed to obtain phytosterols by enzymatic esterification with FFA from rice bran. The literature review shows that immobilized *Candida rugosa* lipase (CRL) on SiO_2_@P (MAA-co-VBC-co-DVB) particles can get 92.1% conversions (64).

In order to simultaneously carry out the hydrolysis of rice bran oil and the esterification reaction with phytosterols, and have the advantages of high efficiency, sustainability, solvent-free process and multiple recovery, Yang et al. proposed a pH-switchable Pickering interface biocatalyst (PIB) system (65). The system was based on amine-functionalized mesoporous silica nanospheres, with PIB as the emulsifier and AYS@HMSS-N (AYS) as the carrier, and the relative conversion rate of phytosterols was maintained at 95%. In the search for greener, more efficient and less consumptive methods with high yields and high activity, numerous studies are now focusing on the application of microbial fermentation in the production of physiologically active secondary metabolites. Numerous studies are now focusing on the application of microbial fermentation in the production of physiologically active secondary metabolites. The microorganism *Moraxella oviswas* , a probiotic that can utilize rice bran oil and selectively degrade phytosterols, and this strain can produce androst-4-ene-3,17-dione, androsta-1,4-diene-3,17-dione, testosterone, and estrone (66). Deodorization is also the key step of rice bran in the process of deacidification. Adopting nitrogen as the vapor extraction gas is more suitable for deodorization, it promotes the formation of phytosterols, reduces the production of phytosterols oxidation product, and improves the quality of oil (67). In addition, the loss of biological activity of rice bran is a concern. Compared to CPE, SE obtained higher levels of aminobutyric acid (97.37 mg/100 g), γ-oryzanol (3829.65 mg/100 g), phytosterols (599.40 mg/100 g), and polyglycol compounds (332.79 mg/100 g) (68). Afterwards, microwave pretreatment (440 W, 2.5 min) of rice bran increased antioxidant activity (0.5-fold), total phenolic content (1.3-fold), flavonoids (0.9-fold), total tocopherols (2.6-fold), total γ-oryzanol (1.6-fold), and total phytosterols (1.4-fold) (69).

## 6. Biological activities and health benefits of phytosterols in rice bran

### 6.1. Anti-cancer efficacy

As the second leading cause of death and morbidity worldwide, cancer generally refers to the uncontrolled cell growth that may spread to other parts of body (70, 71). These abnormal differentiation and proliferation may result in the dysregulated expression of genes involved in cell cycle control (72). The great costs and poor therapeutic effect associated with conventional therapies limit the clinic therapeutic efficacy. Diet has been identified as an important and modifiable risk factor for cancer (73, 74). Therefore, modification in dietary habits that involves the inclusion of functional food components with chemopreventive properties has been identified as a potential strategy for halting or reversing the early phases before the manifestation of a malignancy. Kim et al. confirmed that γ-oryzanol influence of speed in tumor regression via NK activity and macrophages activation and angiogenesis inhibition. Notably, this strategy has almost no side effect (75). Forster et al. investigated the inhibitory effect on human colorectal cancer (CRC) cells growth for the seven rice bran varieties. Significantly, α-tocotrienol, γ-tocotrienol, δ-tocotrienol, and tocopherols showed 1.3- to 15.2-fold differences, respectively. Based on the spearman correlation analysis of the content of γ-tocotrienol and total phenolics were positively correlated with the CRC cells inhibitory effect. Stoichiometric variation in rice bran components and differential effects on colorectal cancer viability merit further evaluation elucidate their role in dietary colorectal cancer chemoprevention (76). These combined considerations indicate that the tocotrienol-rich fraction-based diet with the potential anti-cholesterol and anti-cancer activity could be potentially good candidates in cancer prevention or treatment.

### 6.2. Anti-inflammation activity

Inflammation functions as a protective reaction against multiple detrimental stimuli, potentially leading to tissue damage and infection, by means of targeted production of proinflammatory molecules (77, 78). In terms of duration, inflammation can generally be categorized into two primary classifications: acute inflammation and chronic inflammation (79). Mizushina isolated cycloartenyl *trans*-ferulate from the byproduct after the sake-brewing production. With the remarkably high selectivity for mammalian A, B, and X pol families, *trans*-ferulate has been observed to mitigte the TPA-induced inflammatory response. This finding implies both the byproduct and cycloartenyl *trans*-ferulate hold potential as an effective agent for anti-inflammatory health promotion (80). Akihisa et al. isolated two *trans*-ferulates and four *cis*-ferulates from edible rice bran methanol extract. Results of anti-inflammatory assay revealed that ferulates presented marked inhibitory effect with the 50% inhibition in the range of 0.1–0.8 mg per ear. Moreover, cycloartenol and 24-methylenecycloartanol presented remarkable inhibitory effect with the ID_50_ in the range of 0.2–0.3 mg per ear. Compared with the corresponding ferulates, eight types of free sterols had weaker activity in the range of ID_50_ 0.7–2.7 mg per ear. Taken together, these evidence could facilitate the advanced utilization of rice derived products within the food-related industry (81).

### 6.3. Hypocholesterolemic protective activity

As a chronic cardiovascular disease, atherosclerosis is the develop of narrowing and hardening of the abnormal cholesterol deposits in the inner layers of arteries (82). It is the result of hyperlipidemia and lipid oxidation and is the key factor leading to cardiovascular diseases such as stroke and heart attack in developed countries (83). Correlativity study showed that combination of hypocholesterolemic agents and functional compositions in diets may restrict the risk of developing atherosclerotic lesions, thus, Revilla et al. isolated enzymatically from rice bran, which consist of sterols, tocotrienols, γ-oryzanol, tocopherols, and peptides. The experimental data suggest that this new product may be a good source of functional food ingredient because of the advantage of water solubility, antioxidant and hypocholesterolemic capacity in comparison with rice bran (84).

### 6.4. Skin improvement activity

It is known that skin health is not only significantly caused by overexposure to solar ultraviolet (UV) radiation, but also by contaminant, chemical oxidants, microorganisms, and inflammatory reactions (85). Skin tissue, which consist of the epidermis, dermis, and subcutaneous fat layers, plays a role in maintaining skin barrier function (86). While the damage of skin barrier function has been widely confirmed by an altered stratum corneum integrality, with a consequent increase in trans epidermal water loss and decrease in skin hydration (87). Thus, discovering new dietary bioactive to have a positive influence on skin health conditions is extremely important (88, 89). Earlier, Yasukawa et al. observed that the functional composition from rice bran remarkably inhibited the TPA-induced inflammation with IC_50_ at 0.2–0.3 mg per ear. Amongst 24-methylcholesterol ferulate, sitosterol ferulate, cycloartenol ferulate, and 24-methylenecycloartanol ferulate, cycloartenol ferulate presented the significant inhibition ability of TPA in 7,12-dimethylbenz[a]anthracene-initiated mouse (90). Recently, Vardhani et al. provided the sufficiently potent of black rice bran extract for utilization in the recipe for skin whitening. *In vitro*, the melanin levels reduced at 3- and 30 μM γ-oryzanol doses, specifically. Also, the ethanolic black rice bran extract excellently inhibited tyrosinase activity with an IC_50_ of 74.8% (91). Kobayashi et al. developed a method for producing phytosterol ester extract, which consist of phytosterols, triterpene alcohols, and fatty acids, from byproducts of the refine rice bran oil. Both the normal human fibroblasts cell assay and image analysis results suggested that phytosterol ester extract could have a positive impact on the deteriorated barrier function. Furthermore, the administration of phytosterol ester extract topically resulted in an anti-inflammatory response and enhanced moisturization by augmenting barrier function, consequently mitigating erythema and pore-related issues induced by inflammation (92).

### 6.5. Anti-obesity activity

Obesity has been correlated positively with an increased risk of developing various severe medical conditions, including diabetes, myocardial infarction, cerebrovascular accidents, as well as certain types of carcinomas (93). To take precautions against diet-induced obesity, Fukuoka et al. demonstrated that rice bran-derived, oil-soluble triterpene alcohol and sterol preparation (TASP) enhanced the efficiency of fat and attenuated diet-induced obesity via reducing the postprandial glucose-dependent insulinotropic polypeptide (GIP) content, which indicated that the enriched TASP product may be a promising approach to develop GIP-based obesity-controlling product (94). During energy restriction-induced weight loss in overweight and obese adults without cholesterol-lowering medication intake, Hongu et al. confirmed that plant sterols in rice bran could influence the obesity intervention on lipid profiles. Collectively the findings indicate that overweight and obese adults who followed a diet that was both nutrient-balanced and restricted in calories by 25%, while also using rice bran and plant sterols as supplements, had a notable reduction in their levels of LDL-C and total cholesterol (95).

### 6.6. Anti-diabetic activity

Previous studies have confirmed that dietary supplementation, such as *chlorella vulgaris* (96), carotenoids (97), and salidroside (98), can protect against diabetes mellitus and its complications (99, 100). Nevertheless, the misunderstanding of the molecular mechanisms in regulating diabetes diseases and the potential utilization of nutritional supplementation is currently undergoing comprehensive researches. For example, Kaup and co-workers provided both *in vitro* and *in vivo* validation of stabilized rice bran extract had a concentration-dependent anti-diabetic effect, which was of interest that γ-oryzanol might be a candidate for anti-diabetic effect. Thus, it is reasonable to extrapolate that the prevalence of diabetes or at least a prediabetic situation can effectively retard via rice bran extract intake (101). On the basis of observations from streptozotocin/nicotinamide-induced type 2 diabetes rat model, the utilization of a rice bran oil diet by Chen could block the hyperinsulinemic and hyperlipidemic reaction processes. And the hypocholesterolemic mechanism of rice bran oil might due to the high concentrations of γ-oryzanol and γ-tocotrienol could enhance fecal neutral sterol and bile acid release via upregulating cholesterol synthesis and catabolism (102).

### 6.7. Neuroprotective activity

Neurodegenerative diseases, characterized by progressive loss in selected areas of the nervous system, are becoming one of the increasingly and common global health burdens (103, 104). Particularly in the elderly, aging-related neurological disorders are now the leading source of disability in the world and Parkinson’s disease is the fastest-growing of these disorders. Despite their diverse clinical manifestations, neurodegenerative diseases are multifactorial disorders with standard features and mechanisms such as abnormal protein aggregation, mitochondrial dysfunction, oxidative stress and inflammation. From rice bran and rice bran oil, Zhang et al. demonstrated that campesteryl ferulate, 24-methylenecycloartanyl ferulate, sitosterol, and cycloartenyl ferulate presented dopaminergic neuroprotective ability via the DAF-16/FOXO pathway activation and the inhibition of the apoptotic protein CED-3 expression. In terms of these findings, a significant insight into the potential of four phytosterols as therapeutics in protecting the dopaminergic neurons was provided (105). Thus, this rice bran-based food product might be considered as potential candidates to be used as dietary supplement, helping to prevent the development of the neurodegenerative diseases.

### 6.8. Gastroprotective activity

Peptic ulcers are one of the major human illnesses of the entire gastrointestinal tract, which affecting nearly 10% of the world’s population, among them, 5% suffer from gastric ulcers (106). The imbalance between endogenous aggressive factors and cytoprotective factors lead to the pathogenesis of gastric ulcers, which occur mainly in the stomach and the proximal duodenum (107). Recently, scientific evaluations on different types of phytosterols have been identified and revealed to exhibit potential prevention or cure activities of peptic ulcers. Therefore, the assessment of food-derived components potential as a source of an anti-ulcer agent is still one of the important challenges confronting medicine nowadays. Trinovita et al. obtained rice bran extract by ionic liquid-microwave-assisted method and calculated the effectiveness of rice bran extract as a gastroprotective in ethanol-induced acute gastric ulcer models. Results show that rice bran extract could suppress the ulcer formation by 66.75% and gastric acid levels, and increased the secretion of gastric mucus at the dose of 400 mg kg^−1^ BW. Thus, rice bran extract has potential to be considered as gastroprotective agents from rice bran in gastric ulcers treatments (108).

### 6.9. Anti-allergic activity

Nowadays, there is a renewed interest in developing and discovering anti-allergic phytochemicals for the prevention of allergic diseases, which is a serious health problem worldwide (109, 110). As one of the immune dysfunctions, hypersensitivity reactions can be divided into four general categories based on the mechanism of immunological response: (1) immediate or anaphylactic hypersensitivity mediated by IgE, (2) antibody-mediated cytotoxicity mediated by antibodies of the IgM or IgG classes and complement, (3) immune complex hypersensitivity mediated by IgG or IgM classes, and (4) cell mediated or delayed type hypersensitivities. Recently it has been pointed out that dietary change might contribute to the onset of allergic diseases. For instance, the essential mechanisms of vitamins, flavonoids and related compounds’ anti-allergic action were detailly investigated. In order to reveal interacting points between cycloartenyl ferulate and allergic reaction, Oka et al. building the passive cutaneous anaphylaxis rats model induced by DNP-HAS (111). After cycloartenyl ferulate and γ-oryzanol were injected intradermally with anti-DNP IgE into the dorsal skin of rats, the hypersensitive reaction was attenuated markedly. Overall, the finding regarding anti-allergic activity was mainly contributed by the cycloartenyl ferulate could binding with IgE and ultimately weaken mast cell degranulation.

### 6.10. Anti-microbial activity

In the study of Castanho et al., γ-oryzanol have extracted and quantified from three different rice bran varieties. Among Ketoconazole, Bifonazole, Ballatinao, Maluit, Dinordo, Arabon, Bora, NSICRC9, Azucena, Macarico, and Ceres, not only the Portuguese Ceres presented the highest bacterial inhibition ability, but also all the varieties could inhibit the majority of bacterial and fungal strains. The antimicrobial activity assays showed that Maluit and Dinorado demonstrated the highest fungal inhibition activity. Anti-tumor activity assay showed that Ballatinao with the highest content of γ-oryzanol also presented the greatest capability against all the tested tumor cell lines. Therefore, all the results indicated that rice extracts may be used as the potential candidates od natural antimicrobial and antitumor agents (112).

### 6.11. Immune protective activity

The human innate immune response is of prime importance in host defense system against pathogens, which consists of two branches: innate and adaptive immunity (113). The innate immune system recognizes pathogens and apoptotic cells through particular receptors and responds by activating immune competent. It is clear that recent studies indicate that phytosterols in *Hypoxis* erxtracts might ultimately influence the innate immune system response. By studying the immune protective activity of γ-oryzanol-rich extract from black rice bran, it was found that the enhancement of the phagocytic activity of RAW264.7 macrophages occurred, and the expression level of Toll-like receptor 4 and cluster of differentiation 14 elevated. Moreover, γ-oryzanol-rich extract and γ-oryzanol could promote the production of IL-8, and CCL2, which suggest that γ-oryzanol could be developed as a functional food components for improving the innate immune ability (114). Based on the research form Wilankar et al., tocotrienols possess sufficient immunomodulatory activities in murine lymphocytes. Compared with α-tocotrienol, novel findings are reported on the inhibition effect of γ-tocotrienol was more effectively contributed in cytokine formation and concanavalin A-induced T cell proliferation (115).

## 7. Future research direction

Rice bran oil, with its optimal proportion of fatty acids and rich nutrient content, can be utilized to produce functional blended oils. By incorporating rice bran oil with various other plant oils, the complementary advantages of different oils can create a more comprehensive nutritional profile. This review specifically highlights the presence of phytosterols, beneficial compounds found in rice bran oil, which are typically present in lower quantities in other plant oils. Consequently, the development of blended oils by combining multiple plant oils can significantly enhance the nutritional value of the final product, contributing to a healthier diet. In addition to its uses as a blending oil, rice bran oil can also be used as a frying oil and for the development of healthy food products. Additionally, further research is still needed to fully understand the biological effects of phytosterols present in rice bran oil. The thriving and fast-paced growth of the rice bran industry is expected to bring significant economic benefits and societal value.

## 8. Conclusion

In summary, rice bran oil offers numerous nutritional benefits due to its abundance of phytosterols, making it an excellent choice for the high-value utilization of rice bran, a byproduct of rice processing. Not only does it have the potential for application in functional food products, but ongoing advancements in extraction and processing techniques have resulted in rice bran oil products with outstanding stability and sensory attributes. As a result, this progress not only fuels further exploration and development of rice bran within the food industry but also opens doors for its application in pharmaceuticals, chemicals, and other diverse sectors.

## Author contributions

ZL: Conceptualization, Writing – original draft, Data curation. XL: Data curation, Writing – original draft. ZM: Funding acquisition, Supervision, Writing – review & editing. TG: Funding acquisition, Conceptualization, Writing – original draft.
