# Supplementary figures and images for: Phytosterols in rice bran and their health benefits
Source: Front Nutr. 2023 Oct 12;10:1287405. doi: 10.3389/fnut.2023.1287405 (PMC10600523; doi:10.3389/fnut.2023.1287405)

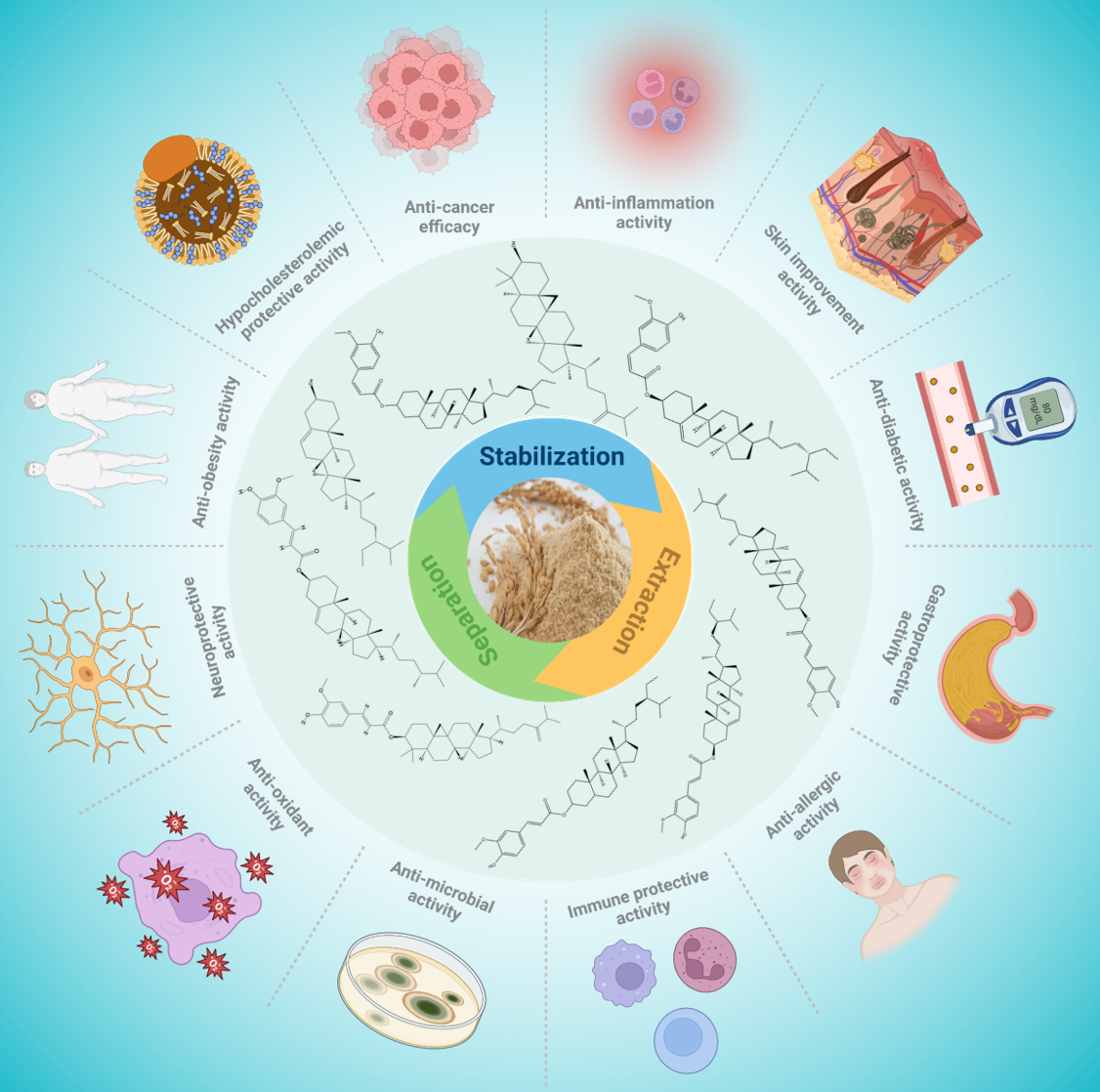

Supplement: Supplementary file 1 [file Image_1.TIF]
